# Acceptability of COVID-19 Certificates: A Qualitative Study in Geneva, Switzerland, in 2020

**DOI:** 10.3389/fpubh.2021.682365

**Published:** 2021-08-17

**Authors:** Vanessa Fargnoli, Mayssam Nehme, Idris Guessous, Claudine Burton-Jeangros

**Affiliations:** ^1^Geneva School of Social Sciences, Institute of Sociological Research, University of Geneva, Geneva, Switzerland; ^2^Division and Department of Primary Care Medicine, Geneva University Hospitals, Geneva, Switzerland; ^3^Faculty of Medicine, University of Geneva, Geneva, Switzerland

**Keywords:** immunity certificates, COVID-19, anti-SARS-CoV-2 serology, social acceptability, qualitative study, public health policy

## Abstract

Immunity certificates related to severe acute respiratory syndrome coronavirus-2 (SARS-CoV-2) have been under discussion since the beginning of the pandemic with conflicting opinions. In order to identify arguments in favor of and against the possible implementation of documents certifying immunity of an individual based on serological testing, we developed a qualitative study in Geneva, Switzerland. The study took place between two lockdowns with a sense of semi-normalcy during summer 2020 in Switzerland but at a time when no vaccine was available and seroprevalence was below 21%. Eleven focus groups with members of the public and 14 semi-structured interviews with stakeholders were conducted between July and November 2020, with a total of 68 participants with an age range between 24 and 77 years. Interviews and focus groups transcripts were coded with the ATLAS.ti CAQDAS. Few participants considered immunity certificates based on serological testing as an acceptable public health measure. Major concerns included the reliability of scientific data related to COVID-19 immunity and serological testing potential re-infection as well as the possibility that the use of certificates could result in deleterious outcomes. Discrimination, counterfeiting, incitement for self-infection, invasion of the private sphere, violation of personal integrity, and violation of medical secrecy were perceived as the major risks. Benefits of immunity certificates were more perceived when in relation to vaccination, and included gains in medical knowledge and protection in certain contexts involving leisure or work-related activities. The consequences of implementing immunity certificates are numerous, and the acceptability by the general population has to be considered when engaging in such policy. Even if the results provide a snapshot of arguments discussed around immunity certificates based on serological testing before the implementation of the COVID-19 vaccine, most of the issues discussed are central in the current debates about vaccination certificates.

## Introduction

“Immunity passport,” “risk-free certificate,” “release certificate,” “immunity certificate,” “antibody(ies) certificate,” “COVID-19 immunity-based licenses,” many terms are employed to name a document aimed to certify immunity of an individual against severe acute respiratory syndrome coronavirus-2 (SARS-CoV-2), the virus causing COVID-19 (1–3). In theory, such a document—often compared with the yellow fever certification—would guarantee that “an individual has been infected and is purportedly immune to SARS-CoV-2” [(3) p. 1595]. Although antibodies and immunity against SARS-CoV-2 are starting to be better understood, they remain uncertain to date (3, 4). In 2020, when no vaccine was available, some countries were considering using a certification status based on anti-SARSCoV-2 as an alternative measure to prevent lockdowns and/or to relax restrictive measures (1, 3–6). Currently, with the COVID-19 vaccine rollout, conversation has intensified over this subject with immunity certificates seen as a solution to free individuals from social restrictive distancing regulations (5). Some governments see in this strategy a way to alleviate public health measures and to pave the way to normalcy, similar to a pre-pandemic state. Therefore, immunity certificates are perceived as useful to avoid additional lockdowns, to further individual freedom and end physical restrictions, to resume activities and restore economic markets by opening workplaces and facilitating travel, to enable close social interactions while mitigating complications from infection especially for some individuals whose work involves close interactions with “vulnerable” people and/or patients, to have access to facilities such as nursing homes, to socialize or even to offer psychological support to individuals, while protecting public health and healthcare services (3, 5).

For both types of certificates (natural or vaccine-acquired immunity), discussions about their implementation are dominated by ethical arguments (7). Indeed, if in 2020 the considerations to implement them were based on seroprevalence testing, in 2021 the debate is centered on vaccine certificates with vaccination programs initiated in many countries. At the time of the study, debates focused only on immunity certificates based on serological testing. Arguments in favor of immunity certificates were to pursue the “least infringement” public health principle or the “least restrictive alternative” for individuals (1, 8). Concerns against their implementation were related to the small number of people with a positive serological test, as evidenced in a Geneva-based study in June and December 2020 (6, 9), the reliability of scientific data to date (the presence of antibodies is to this day not seen as an accurate indicator of sterilizing immunity); equitable and legal challenges such as the risk of discrimination; violation of liberties; falsification; negative impact on behaviors and nonstandard applications (1, 3–5, 8). Additionally, risk-taking by individuals with a positive antibody test may increase the probability of transmission (1, 3, 4, 10). Even the term “immunity” seemed controversial when discussing natural immunity and could generate a “false sense of security” (5). At the time of publication, several of these issues remain unsolved, and discussions shifting toward vaccination certificates present similar arguments.

The objective of this qualitative study is to identify arguments in favor of and against the possible implementation of immunity certificates related to SARS-CoV-2 in Switzerland, and to pave the way to a policy-based discussion. We conducted interviews with stakeholders working in Switzerland and focus groups with members of the Geneva population to collect diverse opinions at a time when the public debate was limited. The study took place in Summer and Autumn 2020, between two lockdowns with a sense of semi-normalcy during summer 2020 in Switzerland, with less emphasis or perception of COVID-19-related pandemic pressure. At that time, the seroprevalence was below 21% (6). When the study was conducted, most governments were not considering immunity certificates yet. No immunity certificates were implemented in any country, and vaccine certificates were not publicly discussed.

## Methods

In June 2020, the ECI study (study on immunity certificates) was started to evaluate the acceptability, feasibility, and utility of immunity certificates in Switzerland. The study was comprised of three consecutive parts with an initial quantitative questionnaire sent to 1,520 participants of a Geneva-based seroprevalence study (SEROCoV-POP study^1^), a qualitative component described herein, followed by a quantitative questionnaire based on the results of the qualitative study.

The qualitative component was conducted between July and November 2020 and explored the acceptability, feasibility, and utility of implementing immunity certificates in-depth and provided insights for the elaboration of a second survey, sent in February 2021 to the same population. In this article, only the findings of the qualitative study are reported^2^.

Fieldwork combined focus groups with members of the population and individual interviews with stakeholders.

Eleven focus groups (4–6 participants each) were conducted with people living in Geneva (*n* = 54). Participants were recruited from the SEROCoV-POP study *via* an online invitation, with over 1,000 email invitations sent. The two inclusion criteria were age (adults of 18 years and older) and language (ability to speak French or English). Individuals who lacked a capacity to consent and those whose physical participation was deemed unsafe to themselves or others (participants who were in self-isolation or quarantine) were excluded. Age was used to construct homogeneous groups as much as possible (one group aged between 24 and 26 years, three groups aged between 30 and 55 years, four groups aged between 41 and 72 years, one group aged between 66 and 77 years, and two mixed groups aged between 25 and 70 years old). This was in order to avoid a too strong polarization of views, which could negatively impact the focus group discussion. All groups were mixed in terms of gender, except one composed of women only.

Fourteen semi-structured interviews were conducted with stakeholders working in Switzerland and engaged in decisions related to the COVID-19 epidemic, including politicians, medical experts, public health experts, economists, entrepreneurs, information technology experts, experts in ethics, and representatives of civil society. They were recruited through a purposive sampling with a list of stakeholders we elaborated on the basis of their expertise in the above fields and connections with the COVID-19 pandemic management. Out of the 22 persons who were contacted, 14 accepted the interview.

Despite time pressure and limited access to the field in the pandemic context, over time, focus groups and stakeholders interviewed provided redundant opinions. We therefore considered having reached data saturation and terminated our fieldwork in November 2020.

For practical and public health reasons, interviews of stakeholders were conducted remotely by videoconference. Focus groups participants could choose between face-to-face and virtual meetings. Four out of the 11 focus groups were held in person and seven by videoconference.

All focus groups and interviews were conducted in French except for one interview in English. Interviews lasted 40 min on average and focus groups 90 min. With permission from participants, all interviews and focus groups were audio-recorded. The interview and focus group guides covered five main topics: (1) acceptability and utility to know the immunity status of an individual; (2) acceptability and utility to know the immunity status of others; (3) divulgation of immunity status of an individual; (4) expected information on the certificate; (5) implementation of an immunity certificate. To initiate the discussion, two scenarios were proposed one where a grand-daughter could only visit her grandmother in a nursing home after providing an immunity certificate and the other where an employer imposed an antibody test on his employee in order to conduct business with customers (see the interview guides, Annex 1).

This study was approved by the Cantonal Research Ethics Committee of Geneva University Hospital, Switzerland (CCER-16-363). An information letter and written consent were signed by all participants prior to conducting the interviews and focus groups. Focus groups participants filled a questionnaire detailing sociodemographic characteristics.

Interviews and focus groups transcripts were coded with the ATLAS.ti CAQDAS (see the codes list, Annex 2). The coding process was conducted in two steps: (1) The interview guide topics provided the initial codes and (2) some inductive codes such as medical secrecy and immunity uncertainty were created from the collected data. A thematic analysis classifying arguments as in favor or against immunity certificates was adopted (11).

## Results

The age of the stakeholders varied between 41 and 74 years, and two-thirds were men. Out of the 54 focus groups participants, 30 were women and 24 men. Their age varied between 24 and 77 years, and the majority had a university diploma (*n* = 34; Table 1). Twenty-nine participants were Swiss, 22 European (outside of Switzerland) and three non-European. Twenty-five participants were employed full-time and 15 part-time, of which 11 were women and 4 men. Most of the participants were married or living with a partner (*n* = 32). The majority of them were living in the city of Geneva (*n* = 44).

**Table 1:** Characteristics of the participants.

	**Interviews of**	**Focus Groups**	**Total**
	**Stakeholders**		
**Gender**			
Men	10	24	34
Women	4	30	34
**Education**			
Lower to middle education^*^	0	20	20
Higher education^**^	0	34	34
Not available	14	0	14
**Age range (years)**			
24–30	0	7	7
31–50	4	18	22
>50	9	29	38
Not available	1	0	1
**Total**	**14**	**54**	**68**

* *Lower to middle education includes compulsory schooling, apprenticeship, maturity, and specialized schools*.

** *Higher education includes university diplomas*.

Arguments against and those in favor of immunity certificates are sequentially presented below, according to the data collected both during focus groups and during individual interviews. We consider these data jointly since there was no major divergence across the two study populations. Their respective extent reflects opinions of interviewees expressed spontaneously, with the interviewer keeping an independent stance. To preserve the anonymity of the participants, only the gender (man or woman) and the age of the participants of the focus groups are specified.

### Immunity Certificate Disadvantages

Arguments against the implementation of immunity certificates were discussed around six main questions, recurrent across the focus groups, and interviews of stakeholders.

#### Is Scientific Knowledge on Immunity Certificate Reliable?

The limited reliability of scientific data related to COVID-19 immunity—including meaning, strength, and duration of the immunity; serological test, mutation of the virus; and potential re-infection—was the main reason brought up to reject immunity certificates. Most of the participants acknowledged that political decisions should not be made on the basis of weak evidence around antibody testing. Immunity certificates were seen as “*irrelevant*,” “*useless*,” and even “*dangerous*” as quoted by some interviewees:

“*For the moment we don't know what it means to be immune, neither for how long, nor against what, [we don't know about] the mutation of the virus, so today it doesn't mean anything and it can't be relevant to have a certificate!” (stakeholder)*.

“*In the current state of knowledge, it doesn't mean you have the antibodies because you've had the disease. And, there's not really a link between having the antibodies and not being contagious either!*” *(36 year old man)*.

#### For Which Purpose(s) Would the Immunity Certificate Be Used?

The majority of participants pointed out the importance to specify the purpose(s) for which immunity certificates could be used, as well as their period of validity prior to implementing them.

Most participants perceived immunity certificates as a tool for discrimination and restriction. The risk of creating a differentiated regime among those who are “positive” and those who are “negative,” those who could travel, work and those who could not was often mentioned. Potential discrimination of those not holding a certificate proving their immunity, who could represent a burden for society, was discussed:

“*The problem is not those who will have it, it is all those who will not have it. The immunity certificate could stigmatize those who are not yet immune, who are a burden to society because we have to protect them*” *(stakeholder)*.

The separation between those who are immune, being the advantaged ones— “the immune-privileged” —and those who are not— “the immune-deprived” —was strongly denounced and perceived as deleterious to social cohesion:

“*This immunity certificate will create a divided society with privileged people and unprivileged ones. We really need more social cohesion instead of a society that consists of two groups of people*” *(56-year-old woman)*.

Immunity certificates were considered mostly as an open door to potential drifts and would set a “*dangerous*” precedent:

“*It's the Pandora's box. Today we open a door for a particular case and tomorrow it becomes a generality and we extend it to any field of health*” *(68-year-old woman)*.

“*For sure it would set a precedent. And it is a really dangerous precedent*” *(47-year-old woman)*.

The fear of the creation of “*ghettos*,” with reference to “*yellow stars*” as well as unequal treatment related to child bearing or the AIDS epidemic, was mentioned:

“*I'm afraid that it could lead to abuses and that history repeats itself. I really don't want to suddenly have a yellow star somewhere on my cloth*” *(54-year-old woman)*.

“*There was also the whole period of AIDS that was largely present in the late eighties. There were very strong questions about people's serological status and their place in society*” *(70-year-old man)*.

Cost and access issues were also seen as possible sources of inequalities:

“*Who pays for it? Is it the State? Is it the individual? Not everyone can necessarily afford such a certificate, even if it is 10 or 15 or 20-Swiss francs. We are not all equal. At the end, it could again create inequalities*” *(55-year-old man)*.

Arguments about potential discrimination and inequalities also led to discussions about the value of an immunity certificate.

#### What Is the Value of an Immunity Certificate?

Its economic value such as professional advantages was often quoted, along additional risks of discrimination and professional difficulties:

“*We're going to say this word of segregation between those who will have financial access to prove to their boss that they can come to work and meet clients and those who won't be able to*” *(58-year-old woman)*.

“*What do we do with all the people who finally didn't have ‘the chance’ to be exposed to the virus and develop antibodies? Are they being penalized in their work? Or in their search for work? Because we can imagine that the next step would be to say: we have a pool of employees but now we are going to recruit new people, do we ask these new people to have a test that confirms their immunity before recruiting them?*” *(50-year-old woman)*.

This risk refers especially to professions involving close face-to-face interactions such as health professionals or teachers:

“*I can already see the excesses where parents will require the teacher to go under... for fear of letting their children go to school. A teacher who is not immunized could be banned from public education! It scares me. Or maybe even in the medical field, would it be a requirement for nurses?*” *(61-year-old woman)*.

Many participants feared that private companies would have access to these data, quoting particularly health insurances using immunity status to increase their premiums.

The potential economic value of the certificate implied risks of falsification and implementation of “*black markets of certificates*” as highlighted in all focus groups and interviews with stakeholders.

“*It would open the door to fake certificates. When there's the job in the balance, that is to say that if the employer can tell you:* ‘*Well listen, sorry but your test is negative, so goodbye*,’ *or I don't know what, they'll put you at 50%, or in partial unemployment or whatever, there's going to be of course false certificates … or into trafficking dates because you were positive* × *time ago and negative afterwards. Well, I mean, the issue of work and employment is important enough so that people find biases and ways to face it*” *(57-year-old woman)*.

The economic value of the immunity certificate was also perceived as an incentive to expose oneself to catch the disease, often expressed as an encouragement to take part in “*coronaparties*.” As one stakeholder said: “*To become immune you have to catch the coronavirus!*” which could lead to intentional contaminations.

This collateral damage was considered as unacceptable both from a societal and from an ethical point of view potentiating the risk of people being voluntarily inoculated with the disease. This risk was seen as an individual and collective danger:

“*The immunity certificate, which would be based on serology, would send a signal that would be the opposite of individual protection. If you want to enter our country, you can only do so if you have exposed yourself to the risk and have had the disease. Somehow this is an incentive to catch it [the virus] in order to benefit from the certificate. So, the certificate is not something that is a solidarity and collective incentive to protect the health of the individual, but it is an individualistic incentive that aims to expose more people*” *(stakeholder)*.

Furthermore, most interviewees insisted that immunity certificates should not be used to clear oneself or “*reward*” those who have caught the disease:

“*People who have a certificate or have had the Covid will have more freedom, will be able to do more things than people who haven't had it and I think that socially it would not be correct and not accepted by a large part of the population*” *(25-year-old woman)*.

“*People who followed the rules, who really paid attention, if they're penalized for not having Covid, it wouldn't be fair either*” *(35-year-old man)*.

#### Is the Certificate Compatible With Already-Implemented Public Health Measures?

The majority of interviewees argued that an immunity certificate could represent a threat to the current health measures that include wearing masks, physical distancing, contact tracing, quarantine of contacts, isolation of cases, and PCR tests. These measures were perceived as “*enough*,” “*better*,” and “*safer*” than serological testing:

“*With all the barrier gestures that we already have, we are obliged to disinfect our hands, to wear a mask, to be not closer than 1.5 m, so there is little risk of infecting anyone. I don't think the immunity certificate will bring a plus. Honestly, No!*” *(55-year-old woman)*.

Several experts emphasized the importance “*to detect quickly infected people and isolate them*” rather than to know the immune status of an individual. For the majority of interviewees, current measures would still remain in place regardless of immunity certificates, emphasizing the futility of this document.

For some stakeholders and members of the population, this document was even seen as a disproportionate measure in relation to other risks related to health such as tobacco and alcohol, reinforced by the low-lethal nature of the COVID-19:

“*It's not acceptable to issue this certificate. It seems to me completely abusive because the average age of death of this disease is about 84 years and the life expectancy is similar. This disease is not a danger that justifies this kind of measures. It seems completely excessive. Yes, it has killed people but it was mostly people who were at the end of their lives and with conditions of connected diseases. For me it's completely disproportionate*” *(54-year-old man)*.

“*Typhus 100 years ago or these kinds of diseases were extremely contagious, there was no treatment during pandemics, I can imagine that coercive measure could be justified. But here, it's a disease that is serious for certain groups of the population, but there are many people who, with or without treatment, are doing very well. This measure is too coercive and is absolutely not justified at this stage. It's not ‘lethal enough’ both in terms of the number of people infected and the risk… well, I'm a little embarrassed to say… the number… the proportion of the population affected… does not seem to justify coercive measures*” *(50-year-old man)*.

Some participants feared that the COVID-19 vaccine would be the next “*forced step*” imposed by the government to obtain an immunity certificate:

“*Isn't there the drift that we're going to have to be vaccinated in order to have this immunity certificate? And we know very well that there are people who don't want to hear about vaccination and that it's a drift where we would be obligated to be vaccinated!*” *(54-year-old woman)*.

For most participants, the immunity certificate did not bring any added value in the current management of the epidemic.

#### How Could (Health) Individual Privacy Be Guaranteed?

Finally, immunity certificates were perceived by the majority of interviewees as an invasion of the private sphere and a violation of personal integrity. Participants worried about the privacy of their health data. These concerns were stronger among focus groups participants and often quoted as the first argument against their implementation:

“*It is still an intrusion into people's state of health. Are we testing women to find out if they are pregnant? Well, I don't know, it's still shocking*” *(64-year-old woman)*.

The risk of the violation of medical secrecy was also often highlighted:

“*It [the certificate] is going to attack the medical secrecy and that's a very big problem because medical secrecy is the basis of medicine. Medical secrecy is extremely important because it allows patients to talk about things that are important to him/her where he/she needs help and to know that his/her doctor is going to listen to him/her and he/she is not going to start making it public everywhere. So, there's already a breach of medical confidentiality and that's a big issue*” *(48-year-old man)*.

For participants, the immunity status could not be kept confidential if it had to be presented in order to gain advantages. It was therefore a sensitive issue as individuals could easily “*lose control*” over the circulation of information. Additionally, long-term effects of the disease, as of yet unknown, could lead to potential further health complications, thus transforming immune status into a sensitive issue in the future:

“*The consequences of this information, in the absolute, may not be dramatic, but even though we don't know very well what it is, let's imagine that we find out something about the virus, for example, that some time later it reappears and all the people who were carriers of the virus develop this or that pathology, there the situation would be very delicate*” *(stakeholder)*.

### Immunity Certificate Benefits

Arguments in favor of the immunity certificate were related to some individual and collective benefits they could provide, but in a regulated context.

#### The Immune Status Is Not or Should Not Be Considered as Sensitive Data

If for the majority of the interviewees, the immune status was considered as sensitive data since it is a medical data, one stakeholder argued the opposite. Indeed, in comparison with the HIV+ status, highly sensitive due to the stigmatization attached to HIV/AIDS, the COVID-19 immunity does not represent any risk of discrimination for this expert who saw “immune-privileged” as a “*natural thing*:”

“*I don't see much danger. There are always people who will cry out for discrimination but personally I don't think it's a real fear. The natural evolution of the epidemic is going to make that there are people who are immune, so they are privileged in a certain sense… there's nothing we can do about it! It's just the way it is!*” *(stakeholder)*.

He added that knowing immunity of an individual could be useful both for immune and for nonimmune individuals. One participant of a focus group highlighted that since the purpose of the certificate was to gain some freedom, it could not be considered as a sensitive data.

“*It's an information that if it's going to be useful, it's not possible to hide it, these two things don't go together*” *(55-year-old woman)*.

If the immune status is not considered by these interviewees as a sensitive data, it is also the case regarding the disease. Indeed, some participants noticed that “*having had the covid*” today is “*something cool*.” Some even mentioned that they had heard colleagues or friends “*being proud of having had the covid*.” This illustrates how COVID-19 individual status is actually easily divulgated in the population and not perceived as stigmatizing.

#### To Know One's Immune Status Is Useful …

##### …for Medical Knowledge

Some stakeholders and participants perceived a medical interest in collecting the serological status of the population. It could serve scientific knowledge for medical research and public health prevention and actions, which suggested that, in that case, the use of immunity certificates was acceptable:

“ *It's interesting if it's used on a large scale and it allows you to implement strategies in terms of risk management for the population. I think scientists would probably need that kind of information*” *(64-year-old woman)*.

##### … for Some Categories of People

Some interviewees considered the implementation of immunity certificates acceptable for some categories of people such as healthcare workers to treat patients:

“*I believe that an immunity certificate might not be so bad in the medical field. For example, nurses who have a certificate, it is better that they take care of patients who have Covid*” *(56-year-old woman)*.

A few participants stressed the importance to protect “*vulnerable people*,” referring mostly to the elderly and often in line with a personal situation.

##### …for Some Transactions or Activities

For some participants, immunity certificates should serve as a “*facilitator*” or a “*transactional tool*” to resume some activities such as traveling, being the most systematically quoted example. In that case, certificates were seen as a document that could alleviate quarantine, masks, or PCR testing. However, the same interviewees specified that certificates should not be asked for all activities:

“*You have to travel for your business, you'll travel with more ease than the others who don't have it. Some countries ask to have a Covid test done 72 h in advance and if you have the immunity certificate you could pass easily*” *(55-year-old woman)*.

#### Acceptable Only in Relation to Vaccination

Some interviewees were in favor of immunity certificates only if related to vaccination, as quoted by this stakeholder:

“*For me as long as it's not associated with a widely available vaccine, it's useless. It has to be in the context of wide availability of the vaccine, for me that's the way to start. So, the question you're asking is the right one but, in a vaccine-based temporality*” *(stakeholder)*.

Because vaccination relies on a personal choice, i.e., a voluntary medical act and not the “*chance*” of having caught the disease, immunity certificates related to vaccination appeared more ethically acceptable:

“*I'm not 100% against it, but if it's based on antibody tests, then people don't have the choice to have the certificate or not. You don't choose to have Covid and to be immunized, whereas if it was with a vaccine then in that case you have the choice*” *(25-year-old woman)*.

However, interviewees stressed out that when the vaccination would be available, it should remain optional and affordable to everyone.

#### Implementation Framework

Finally, all participants agreed that if immunity certificates were to be implemented, a strong legal framework was needed in order to ensure that certificates could not be imposed by anyone in an unregulated manner. Criteria should be defined especially regarding the purpose(s) and duration of the certificates. Indeed, certificates should be limited in time, and data should be destroyed after a set duration. These documents should be issued by legitimate and recognized authorities. Appropriate authorities according to participants were at the national level, the Federal Office of Public Health (FOPH), and at the international level, the World Health Organization (WHO). Finally, an expert stressed out that the data should be decentralized to guarantee data security.

## Discussion

This exploratory study is the first qualitative research incorporating opinions of the general population and of stakeholders on immunity certificates. The purpose of this article was to discuss the acceptability to issue a document certifying immunity of an individual against SARS-CoV-2 based on serological testing and its implementation. Qualitative studies do not aim at providing generalizable results. Nevertheless, our study allows us to identify different arguments proposed by stakeholders and members of the population, at the time of the study.

Consistent with other studies and according to the current state of scientific knowledge, disadvantages outweighed the perceived benefits (1, 2). At the time of the study, few participants considered immunity certificates as an acceptable public health measure due to the limited reliability of scientific data. The majority of stakeholders agreed that political decisions should not be made on the basis of serological testing.

Opinions in our study ranged from a light acceptance to total rejection, including ambivalent positions, favoring the use of immunity certificates only in specific cases to protect “vulnerable” populations or contexts such as traveling. We did not notice differences among women and men or among different age groups. Opinions were consensual between stakeholders and members of the public. Nevertheless, medical experts discussed vaccination certificates as a future option more than other participants.

Arguments in favor of immunity certificates were based on the nonsensitive nature of this particular health data. It was perceived as useful for medical knowledge, some categories of people, or personal interest (psychological reassurance). Some saw it as a facilitator to resume specific activities. Immunity certificates appeared more acceptable if based on vaccination status, considered a voluntary act.

Conversely, arguments against immunity certificates stressed the limited reliability of the data, serological tests, immunity (interpretation, duration), and potential mutations of the virus. Major concerns raised were creating discrimination and inequalities between those who are immune—the “immune-privileged” —and those who are not—the “immune-deprived.” It could provoke negative behaviors such as encouraging individuals to catch the disease (intentional or self-infection) or counterfeiting documents. Certificates were seen as counterproductive to fight the spread of the disease; and as violating individual privacy and liberties when these were fundamental to preserve for the majority of the interviewees.

The main paradox quoted was that immunity certificates “incentivise infection” (3) rather than prevent them. Current measures were perceived as sufficient to fight the epidemic in Switzerland, and immunity certificates could undermine prevention efforts according to interviewees. Certificates could contribute to increase inequalities in relation to the costs and access to tests and certificates (4, 8). For interviewees, sanitary measures need to respect and ensure treatment equality and tests and antibody certificates should not “become a luxury of the rich” (5). Access to immune status information by private organizations was also questioned, especially the impact it could have on the premium of health insurances. Decentralized digital identity was perceived for one stakeholder as the best privacy preserving system. This statement is in line with Gruener (4) and Hicks et al. (5).

For some participants, especially in reference to HIV/AIDS, COVID-19 might not be stigmatizing yet because anyone could catch it, but immunity certificates could create stigmatization especially due to the fact that it would be accessible to a minority of individuals. In line with other studies (1, 3), this paradox of dividing the society based on the immunity status appeared unacceptable to all participants.

Employment, which has been greatly impacted by the pandemic, should not be linked to immunity certificates. Indeed, as mentioned by interviewees and in line with some studies, “employment decisions, such as hiring and firing, cannot be made on the basis of health status” [(4) p. 22]. An employer should not be able to impose antibody testing and immunity certificates to his/her employees in order to be able to resume work (10).

Persad and Emanuel (8) concluded that “immunity-based licenses have the potential to help realize important values, including enhancing the liberty of individuals who have been infected with COVID-19 without worsening the situation of those have not been infected, maximizing benefits to individual and society by allowing immune people to engage in economic activity, and protecting the least advantaged by allowing safer care for vulnerable populations” (p.2242). However, all these arguments were perceived discriminatory by the majority of the participants and stakeholders of our study. Finally, interviewees feared that people might be more willing to make compromises and be tempted to “*accept this measure and others*” if they ensured a return to a “*normal life*.” Indeed, overtime, the COVID-19 “fatigue” (12), especially when confronted to recurrent lockdowns and relaxation measures, is strong and negatively affecting the majority of individuals according to participants.

These results provide a snapshot of arguments discussed around immunity certificates based on serological testing before the implementation of COVID-19 vaccination, when evidence on immunity was still provisional and public information limited. Today, digital COVID certificates are being implemented across European countries (13), taking into account, immunity,vaccination and testing for the presence of the virus during acute infection. These certificates are likely to change the population expectations and attitudes as reported elsewhere (14). Despite this new context, the majority of the arguments raised in this study are still relevant in Switzerland and in other countries. Most of the issues discussed above are indeed at the heart of current debates about vaccination certificates. They include scientific evidence about the strength and duration of immunity (natural and vaccine induced immunity); the different purposes that immunity certificates can fulfill; ethical issues such as discrimination and privileges; the competition with implemented measures such as physical distancing and masks and security of personal health data.

### Limitations

This study has some limitations. The interviews took place between two lockdowns with a sense of semi-normalcy during summer 2020 in Switzerland. They provide information specific to that time period, and we can expect that opinions will be different at later stages of the pandemic. The implementation and expansion of COVID-19 certificates are also likely to impact the attitudes of the population (15). This context might have influenced the participation of certain individuals encouraged to share their opinions that could have been influenced by pandemic “fatigue” or “anxiety” stemming from the overall conditions. Some potential participants might have been discouraged by the technology not managing virtual applications (a pre-focus group videoconference testing session was proposed to all participants if requested, in order to eliminate technology resistance or barriers). A selection bias is possible with participants having higher levels of education than the general population. We faced some difficulties to recruit “young” participants, with only six participants in-between 24 and 26 years. The majority of the focus groups interviewees are in-between 50 and 69 years (*n* = 29). Some interviewees made some inquiries on the topic prior to participating, showing their interest and commitment.

In conclusion, the COVID-19 pandemic has severe negative effects both at the collective (economic, political, and societal) and at the individual levels. However, there are several potentially adverse consequences of immunity certificates related to serological testing and the acceptability by the population has to be considered before any potential implementation. With vaccine rollout, vaccination certificates are now at the forefront of academic, political, economic, and medical discussions. Vaccines could answer some of the questions raised in this study, including the interpretability of immunity, its duration, and effectiveness; however, some questions remain around mandatory implementation of certificates, access and rights, freedom of choice as well as feasibility. Therefore, the empirical findings of this unique qualitative study conducted in 2020 bringing together opinions of stakeholders and members of the general public can clearly inform the current discussion about the implementation of vaccination certificates. They offer pioneer results before the development of large public and political discussion on this topic.

Engaging civil society in answering these questions is paramount, especially when such measures will affect the populations in general. Several participants thanked us for being called upon to express their opinions, wishing to be more involved and consulted about these subject matters, revealing that the management of this epidemic could benefit from conducting a public transparent and open dialog with the population.

## Data Availability Statement

The raw data supporting the conclusions of this article will be made available by the authors, without undue reservation.

## Author Contributions

VF conducted all interviews with the stakeholders and nine focus groups, assisted in the qualitative protocol development, developed the qualitative tools (interview guidelines and documents), analyzed and interpreted the qualitative data, and wrote and finalized the manuscript. MN and IG were the main investigators of ECI study (CCER-16-363) and provided essential comments and correction to the final manuscript. CBJ conducted two focus groups, developed the qualitative protocol, and provided essential correction to the final manuscript. All authors participated in the recruitment of the participants and read and approved the final manuscript.

## Conflict of Interest

The authors declare that the research was conducted in the absence of any commercial or financial relationships that could be construed as a potential conflict of interest.

## Publisher's Note

All claims expressed in this article are solely those of the authors and do not necessarily represent those of their affiliated organizations, or those of the publisher, the editors and the reviewers. Any product that may be evaluated in this article, or claim that may be made by its manufacturer, is not guaranteed or endorsed by the publisher.

## Footnotes

^1^Held by the University Hospitals of Geneva (HUG), the SEROCoV-POP study is a population-based serosurvey that took place in the population of Geneva, Switzerland.

^2^The results of the initial quantitative component of study are presented in the article by Nehme et al. (7).
