# Supplementary material for: Acceptability of COVID-19 Certificates: A Qualitative Study in Geneva, Switzerland, in 2020
Source: Front Public Health. 2021 Aug 17;9:682365. doi: 10.3389/fpubh.2021.682365 (PMC8416097; doi:10.3389/fpubh.2021.682365)
Supplement: Supplementary file 1 [file Data_Sheet_1.docx]

# Annexes

Annex 1: Interview Guides

**1.1 Interview guide for stakeholders**

***Immunity Certificate implementation***

1. In general, would you support the implementation of an immunity certificate for all?
2. If yes: could the certificate be considered as a "preventive measure" against a risk of infection by SARS-CoV-2 in the future (at the same level as current measures? Is it more important or less important than wearing a mask for example)?
3. Do you see any significant danger to consider before the implementation of the certificate?
4. From your point of view, who should implement it (who should be responsible for it)?
5. Shall the certificate be mandatory or optional?
   - 1. If the certificate is mandatory: shall it be for everyone or only for some specific individuals? If yes, for whom?
     2. If the certificate is mandatory: what are the reasons?
6. Who shall be in charge of the costs of the certificate?

***Immune Status Disclosure***

1. In general and from a public health perspective, do you think that the status of particular individuals should be disclosed under certain conditions? (“targeted individuals”, “vulnerable individuals”)
2. Who should have access to this information?
3. On the contrary, who should not have access to this information?

***Information and data confidentiality***

1. If a certificate is to be implemented, what kind of information should it include? Why this information in particular?
2. What kind of information should absolutely not be included in the certificate?
3. How would you like the confidentiality of the data be ensured?
4. Who shall be responsible to ensure the confidentiality of this data (physicians, hospital, laboratories, the Federal Office of Public Health…)

***Conclusive questions***

1. If a certificate is to be implemented, would you like to have one for yourself? If yes, for what reasons?
2. Why did you accept to participate to this study (motivations) ?
3. Would you like to add something else ?

**We thank you for your participation**

**1.2 Interview guide for the focus groups**

Two scenarios were used to initiate the discussion, they were followed by questions to be asked if not covered during the discussion with the scenarios

***1st part: Scenarios***

I will present you 2 scenarios that we will discuss together:

**Scenario 1**:

Lea wants to visit her grandmother in a nursing home. The facility has recently implemented a new rule according to which anyone visiting must show an immunity certificate to protect those residing in the nursing home as they are at risk of complications from COVID-19 due to their age.

- *What do you think about this situation ?*
- *What would you advise Lea ?*

**Scenario 2 :**

Simon holds an important position in a large company that employs over a thousand people. He frequently travels and meets with clients. His employer is particularly alarmed by the pandemic situation and wants all employees to be tested regularly. He heard about an immunity certificate that he would like to make mandatory in his company. Simon feels that his boss is overreacting and sees this measure as an infringement on his freedom. However, he fears losing his job if he objects. He remains convinced that this information is confidential, an opinion shared by some colleagues. He is especially concerned that the certificate will be circulated and that the data will be accessible to anyone.

- *What do you think about this situation ?*
- *What would you advise Simon?*

***2nd part: Questions***

***Acceptability, utility, disclosure***

1. For what purpose should people know their immunity status and for which reasons?
2. In general, for which reasons might people other than yourself be interested in knowing your immunity status?
3. Would you like to know the immunity status of other people? [For example, your partner, co-worker, etc.].

*If yes : Who in particular and why ?*

*If no : Why (reasons to list)* *?*

1. Do you think that this information shall remain confidential or not necessarily?
2. Would you accept easily to disclose your COVID-19 immunity status?

*If yes : To whom in particular and for what reasons ?*

*If no : Why (reasons to list) ?*

1. What are the advantages to disclose your immunity status (probes: travel, specific activities, work)?
2. What are the disadvantages or risks to disclose your immunity status (probes: discrimination,...) ?
3. In general and from a public health perspective, do you think that the status of particular individuals should be disclosed under certain conditions? (“targeted individuals”, “vulnerable individuals”) ?
4. Who could have access to this information?
5. On the contrary, who shall not have access to this information?

***Information and data protection***

1. If a certificate is to be implemented, what kind of information should it include? Why this information in particular?
2. What kind of information should absolutely not be included in the certificate?
3. How would you like the confidentiality of the data be ensured?
4. Who shall be responsible to ensure the confidentiality of this data (physicians, hospital, laboratories, the Federal Office of Public Health…)

***Immunity Certificate implementation***

1. In general, would you support the implementation of an immunity certificate for all?

*If yes: Could you give me the major reasons why you are in favor of the implementation of the certificates (or remind me if already discussed)*

*If no: Could you give me the major reasons why you are against the implementation of the certificates (or remind me if already discussed)*

1. Do you see any significant danger to consider before the implementation of the certificate?
2. From your point of view, who should implement it (who should be responsible for it)?
3. Shall the certificate be mandatory or optional?

*If the certificate is mandatory: shall it be for everyone or only for some specific individuals? If yes, for whom? What are the reasons?*

*If the certificate is optional: what are the reasons?*

1. Who shall be in charge of the costs of the certificate?

***Conclusive questions***

1. In conclusion, what does it mean for you “to be immune”?
2. Why did you accept to participate to this study (motivations) ?
3. Would you like to add something else ?

**We thank you for your participation**

Annex 2 : Codes List

• Certificate Opinions (general opinions not requested during interview)

• Data confidentiality

• Cost of the certificate

• Status disclosure

• Immunity certificate: optional or mandatory

• False protection

• In favor of the implementation of an immune certificate (when asked)

• Against the implementation of an immune certificate (when asked)

• Immunity uncertainty

• Information needed in the certificate

• Information prohibited in the certificate

• Virus mutation

• Not acceptable to inform about immunity status

• Acceptable to inform about immunity status

• Who is responsible to implement certificates and why?

• Scenario 1 : Opinions in favour

• Scenario 1 : Opinions against

• Scenario 2: Opinions in favour

• Scenario 2: Opinions against

• Medical secrecy

• Meaning of "being immune"

• Utility/Purpose to know one's immune status

• Utility/Purpose to know others' immune status

• COVID-19 pandemic experience
